# Towards a more realistic anthropomorphic chest phantom using 3D‐printed and cork‐integrated components

**DOI:** 10.1002/mp.17956

**Published:** 2025-07-15

**Authors:** Joost F. Hop, Ivan Dudurych, Thom R. G. Stams, Geertruida H. de Bock, Rozemarijn Vliegenthart, Marcel J. W. Greuter

**Affiliations:** ^1^ Department of Radiology University Medical Center Groningen Groningen the Netherlands; ^2^ Department of Epidemiology University Medical Center Groningen Groningen the Netherlands

**Keywords:** anthropomorphic thorax phantom, computed tomography, lung cancer, pulmonary nodules, quality assurance, 3D‐printing

## Abstract

**Background:**

Thorax phantoms for computed tomography (CT) imaging often lack realistic lung parenchyma and bronchovascular anatomy. To improve anatomical accuracy, 3D‐printed chest phantoms have been developed as more realistic alternatives to existing models.

**Purpose:**

To evaluate whether an in‐house developed anthropomorphic phantom insert realistically represents the anatomical structures and attenuation characteristics compared to the original phantom and human CT data.

**Methods:**

The anthropomorphic chest phantom “Lungman” was modified by integrating a 3D‐printed insert, cork‐based lung parenchyma, and lung nodules. The phantom was scanned on a CT system and evaluated using qualitative and quantitative CT analyses, comparing attenuation values and histogram distributions to human CT data. Subjective radiologist assessments were conducted to compare anatomical realism between the modified and unmodified phantom.

**Results:**

Qualitative assessment of CT value distribution showed strong similarity between the modified phantom and human lung parenchyma, although the radiodensity characteristics of the 3D‐printed bronchovascular insert still require further refinement. Quantitative analysis confirmed that the modified phantom's parenchymal attenuation (−854 Hounsfield unit [HU]) closely matched human lung parenchyma (−872 HU, *p* > 0.05), whereas the unmodified phantom showed lower attenuation (−997 HU, *p* < 0.05). However, the bronchovascular insert showed lower attenuation than human vasculature (−41 HU vs. 42 HU, *p* < 0.05). Radiologists rated the modified phantom higher for anatomical realism.

**Conclusions:**

This study presents a modified, anthropomorphic thorax phantom that integrates realistic 3D‐printed bronchial and vascular structures, lung parenchyma, and lung nodules.

## 1 INTRODUCTION

With advancements in computed tomography (CT) systems, both in hardware and software components, frequent validation of the performance of new CT technology is required.^1^, ^2^ Medical imaging phantoms are designed to mimic the human body's properties, allowing for the testing and improvement of imaging devices used in the diagnosis of diseases. They provide a consistent and repeatable standard for testing without exposing human individuals to radiation.^3^ Phantoms have been shown to be essential in the optimization of CT scan protocols, such as protocols for lung cancer screening.^4^ Typically, phantoms used for chest CT protocol optimization incorporate structures representing the heart, lungs, ribs, and features like lung nodules, which allows for simulating and evaluating diagnostic accuracy in lung cancer imaging.^5^

A variety of anthropomorphic chest phantoms have been employed to optimize CT protocols.^4^, ^6^, ^7^ However, commercially available anthropomorphic chest phantoms have been critiqued for their unrealistic representation of the geometry and structure of human lung tissue.^8^

To overcome the limitations of currently available chest phantoms, various efforts have been made to develop more realistic, 3D‐printed alternatives.^9^ 3D‐printed phantoms can be directly derived from patient data, making them more anatomically accurate and personalized.^10^, ^11^, ^12^ However, despite recent advancements in 3D‐printing, these phantoms still have limitations. One of the primary challenges is achieving realistic attenuation of tissues like the lungs with materials that accurately mimic tissue properties.^13^ Even advanced, commercial 3D‐printed phantoms do not realistically represent lung parenchyma and print full scale models representing the whole thorax.^10^ Foam has been used to simulate healthy lung tissue, but it has a significant drawback: once molded, foam is neither deformable nor customizable to incorporate various lesions.^14^, ^15^ As an alternative, chest phantoms with cork to represent lung parenchyma have been created for radiotherapy^16^ and pediatric CT,^17^ offering low radiodensity values similar to human tissue between –950 and –700 Hounsfield units (HUs).^18^ However, the cork used in these phantoms is rigid and non‐deformable, making it unsuitable for customization with features like lung nodules.

The anthropomorphic chest phantom “Lungman” (PH‐1, Kyoto Kagaku, Co., Ltd, Kyoto, Japan) is a commonly used phantom for chest CT studies.^19^, ^20^ It offers a fairly realistic simulation of the human thoracic anatomy. However, it lacks in vivo like lung parenchyma and bronchial airways, which limits its anatomical realism.^21^ While the phantom allows for the placement of artificial lung nodules, these need to be attached directly to dense vascular structures or the chest wall, potentially affecting nodule evaluation.^22^, ^23^ Therefore, in the research presented here, we modified this phantom by integrating a 3D‐printed bronchovascular insert, lung parenchyma composed of loose cork granules, and artificial lung nodules, a combination not previously reported in a single phantom model.

The aim was to evaluate whether the modified phantom offers a more realistic representation of the anatomical structures and attenuation characteristics of human lung tissue compared to the unmodified commercial phantom. This evaluation involved an objective comparison of CT attenuation values and histogram profiles between the modified phantom, the commercial phantom, and a clinical reference scan. Additionally, the anatomical realism of both the modified and commercial phantoms was subjectively assessed by two experienced radiologists.

## 2 METHODS

### 2.1 Commercial phantom

**FIGURE 1:**
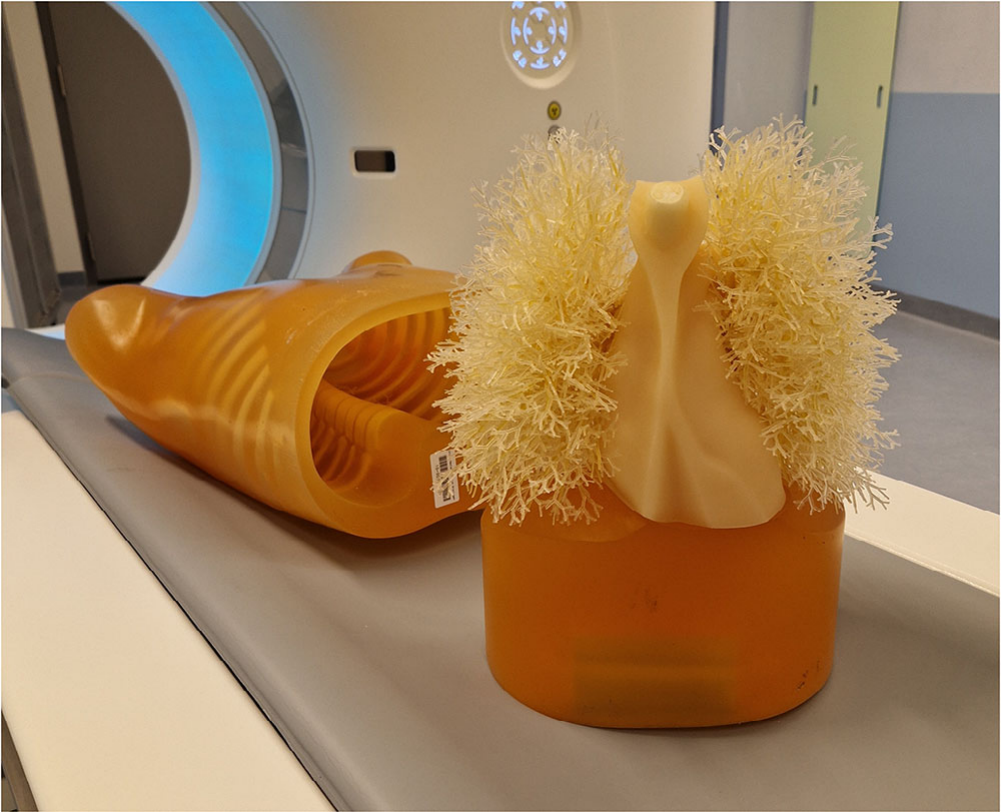
The original anthropomorphic chest phantom “Lungman” consisting of a mediastinum, spine and ribs, pulmonary vasculature, and an abdomen block.^21^

The anthropomorphic chest phantom Lungman was used, which is a life‐size anatomical model of the human thorax, representing an average 55‐year‐old Japanese man (65 kg, 168 cm). Its dimensions were 43 × 48 cm, with a chest girth of 94 cm, an approximate weight of 18 kg, and a lung volume of 4.41 L. The internal components of the phantom consist of a mediastinum, spine and ribs, pulmonary vasculature, and an abdomen block (Figure 1). The soft tissue components were manufactured using urethane‐based resin with a density of 1.06 g cm^−^^3^.^21^

For this study, the pleural cavity of the phantom was used to house a custom‐made insert. The original, commercial phantom is referred to as the unmodified phantom, while the version containing the custom‐made insert is referred to as the modified phantom.

### 2.2 Phantom development

#### 2.2.1 Selection of the human CT scan

For the development of a custom 3D‐printed insert and to serve as a reference for phantom comparison, a CT scan of a participant from the Imaging in Lifelines (ImaLife) study was retrospectively selected.^24^ For this, lung‐healthy individuals were automatically analyzed to find the closest match in lung shape and volume to the Lungman phantom.^25^ Candidate lung shapes were segmented and overlaid on the segmented Lungman phantom cavity using the SimpleITK library.^26^ This step aligned the human anatomy spatially with the phantom's internal space. The top 10 matches were visually inspected for orientation, fit, content, and shape. The closest match was a 52‐year‐old healthy female (64 kg, 167 cm) with normal spirometry, a lung volume of 4.03 L, and an airway volume of 0.12 L.

The ImaLife study was approved by the medical ethics committee of the University Medical Center Groningen, the Netherlands, and all participants provided written informed consent.

#### 2.2.2 Mediastinum, airway, and vessel insert

**FIGURE 2:**
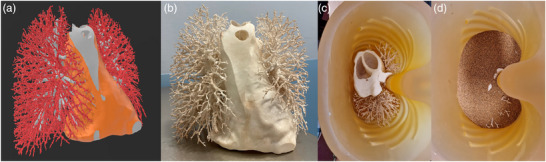
(a) Combined 3D‐model of mediastinum (orange), bronchi (gray), and vessels (red) in preparation for 3D‐printing and (b) the 3D‐printed insert. (c) A caudo‐cranial view of the insert placed inside the phantom's thoracic cavity and (d) cork granules that were poured into the phantom.

The airways, vessels, and mediastinum from the selected human CT scan were segmented using the following methods: airway segmentation was performed using a previously described method and automatically converted into 3D‐meshes;^27^ vessel segmentation was performed with TotalSegmentator;^28^ and the mediastinum (including the heart, aorta, pulmonary trunk, and surrounding tissue) was segmented using 3D Slicer.^29^ These three segmentations were combined and cropped in 3D Slicer to fit within the thoracic phantom cavity (Figure 2a).

The insert was printed using a stereolithography printer (Oceanz B.V., Ede, Netherlands) with Polyamide 12 powder (density 0.93 g cm^−^^3^, minimum wall thickness 0.7 mm) (Figure 2b). The mediastinum was hollowed to a 4 mm wall thickness to prevent warping and overheating during printing. Post‐processing involved powder removal using compressed air followed by an ultrasonic bath in water. The 3D‐printed bronchovascular insert was placed inside the phantom's thoracic cavity (Figure 2c).

#### 2.2.3 Lung parenchyma

To replicate the attenuation values of healthy lung parenchyma, CT scans were performed to evaluate CT values and distribution uniformity of cork granules of varying sizes (0.5–1 mm, 2–3 mm, 3–5 mm; Korex International Sp. z o.o., Radom, Poland). The granules were placed in thin plastic bags and positioned within the Lungman phantom cavity. Scans were conducted at 120 kVp, yielding mean CT values ± standard deviation of –885  ±  21 HU, –870  ±  72 HU, and –927  ±  57 HU for the respective granule sizes. In addition, the density and homogeneity of each cork size were visually assessed in the CT images to check whether the granules formed a compact and uniform volume without air pockets, similar to healthy lung parenchyma. Based on both the measured CT values and visual assessment, the 0.5–1 mm cork granules were selected as the most suitable for mimicking human lung parenchyma. To aim for uniform internal distribution within the phantom, the selected granules were poured into the thoracic cavity in successive layers, with thorough shaking after each layer (Figure 2d).

#### 2.2.4 Lung nodules

**FIGURE 3:**
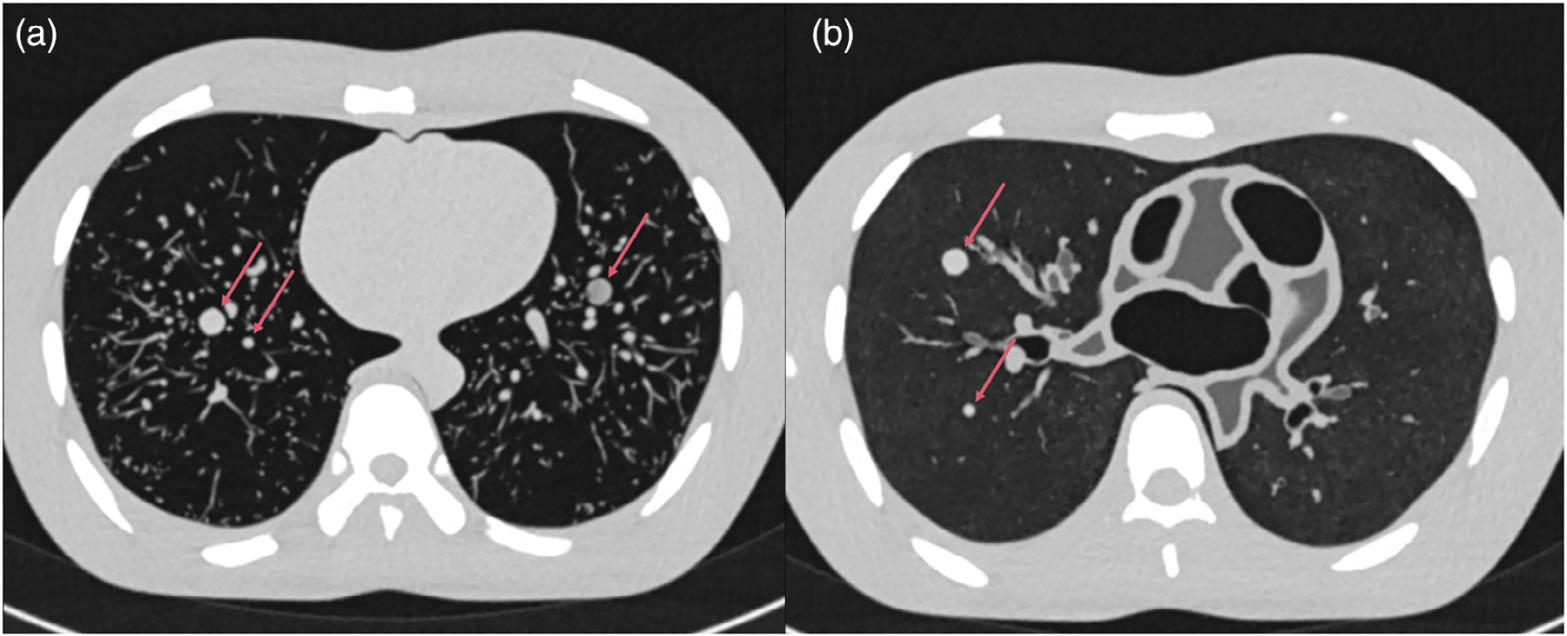
Axial CT images of the (a) unmodified phantom and (b) modified phantom with artificial lung nodules, indicated by the red arrows. The display window level and window width were set to −400 and 1500 HU, respectively.

To compare the realism of lung nodule appearance in the modified and unmodified phantoms, eight spherical artificial lung nodules, described in a prior study, were placed within the thoracic cavity.^30^ The nodules varied in diameter (3, 4, 6, and 10 mm) and radiodensity (−209  ±  25 HU and 66  ±  12 HU), as determined in CT images acquired at 120 kVp tube voltage. In the unmodified phantom, the nodules were inserted by positioning them between branching vessel structures to physically secure them (Figure 3a). In the modified phantom, the nodules were placed between successive layers of cork, positioned between the bronchovascular structures (Figure 3b).

### 2.3 Phantom evaluation

#### 2.3.1 CT protocols

The human CT scan was acquired according to the Imalife study protocol using a third generation dual‐source CT scanner (SOMATOM Force, Siemens Healthineers). Images were acquired at a tube voltage of 120 kVp, a quality reference tube current time product of 20 mAs, a pitch factor of 3.0, and a rotation time of 0.25 s. The volumetric CT dose index (CTDI_vol_) was 0.94 mGy. Image reconstruction was performed with a pixel matrix size of 512 × 512, a field of view of 350 mm, a slice thickness/increment of 1.0/0.7 mm, filtered back projection, and a medium‐sharp body kernel, Br40.

Scanning of the modified and unmodified phantom was performed on the same CT scanner. To ensure that image noise did not dominate the comparison between the phantom's internal structures, a clinical thorax protocol with an increased tube current‐time product, relative to the Imalife screening protocol, was used. The unmodified phantom was scanned at a single tube voltage of 120 kVp, while the modified phantom was scanned at eight different tube voltages: 70 to 140 kVp in 10 kVp increments. The quality reference tube current‐time product was set to 40 mAs. Other acquisition parameter settings included detector collimation of 192 × 0.6 mm, a pitch factor of 0.6, and a rotation time of 0.5 s. The CTDI_vol_ values ranged from 0.50 mGy at 70 kVp to 2.12 mGy at 140 kVp. Image reconstruction was performed with a slice thickness/increment of 1.0/0.7 mm, a pixel matrix size of 512 × 512, a field of view of 300 mm, iterative reconstruction ADMIRE at strength level 3, and a medium‐sharp body kernel, Br40.

#### 2.3.2 Image analysis

Qualitative assessment of the distribution of CT pixel values in the lung areas of the modified and unmodified phantom compared to human CT images was performed.^10^ Normalized histograms representing the frequency of each CT value relative to the total pixel area were generated by placing regions of interest (ROIs) with areas the size of the left and right lung fields of the phantom and human CT scans, acquired at 120 kVp tube voltage, using ImageJ software.^31^ Histograms were computed and combined for 17 adjacent image slices from the upper, middle, and lower sections of the phantom and human scans. The histograms ranged from −1024 HU to 250 HU with a bin size of 5 HU.

To assess radiodensity in pulmonary structures, 10 circular ROIs of 6 to 52 pixels, depending on anatomy size, were defined in the upper, middle, and lower parts of the phantom and human CT scans. These ROIs were placed in areas representing lung parenchyma and pulmonary vasculature. CT values and corresponding standard deviations were measured from the ROIs. Statistical comparisons between the phantoms and human CT values were conducted using the Wilcoxon signed‐rank test. To evaluate the energy dependence of the materials used to replicate the lung parenchyma and bronchovasculature in the modified phantom, CT values were compared across tube voltages using the pairwise Mann–Whitney U tests with Bonferroni correction for multiple comparisons. Statistical analyses were conducted in R (version 4.1.0, R Foundation for Statistical Computing, Vienna, Austria).

As a subjective assessment, two radiologists (RV and TS) with 19 and 6 years of thoracic imaging experience, respectively, reviewed the 120 kVp CT images of the unmodified and modified phantom. To reduce direct comparison and recall bias, the readers first reviewed the images of the unmodified phantom. After a 3‐week wash‐out period, they reviewed the images of the modified phantom. They provided feedback on anatomical realism and overall image quality using a 5‐point Likert scale (1 = very poor, 2 = poor, 3 = acceptable, 4 = good, 5 = very good). Anatomical realism was evaluated based on the representation of bronchial structures, vessels, lung parenchyma, and the appearance of lung nodules. Overall image quality referred to the levels of contrast and noise in the acquired images.

## 3 RESULTS

**FIGURE 4:**
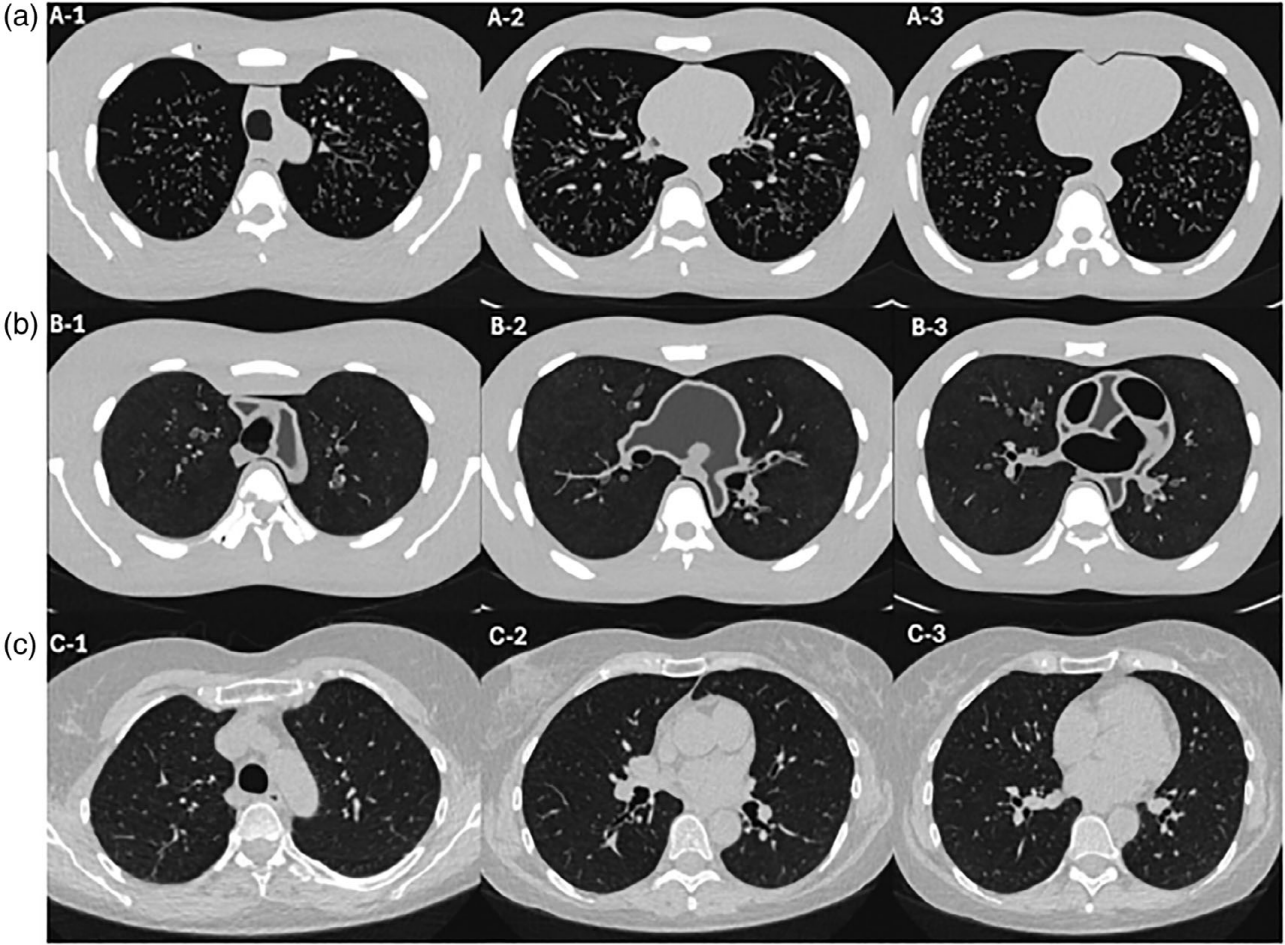
Axial CT images of the unmodified phantom (a), modified phantom (b), and human scan (c) in the upper (1), middle (2), and lower (3) scan sections. The display window level and window width were set to −400 and 1500 HU, respectively.

Modification of the original Lungman phantom involved the addition of patient‐specific bronchial airways and lung parenchyma, features absent in the unmodified version (Figure 4).

### 3.1 CT value distribution

**FIGURE 5:**
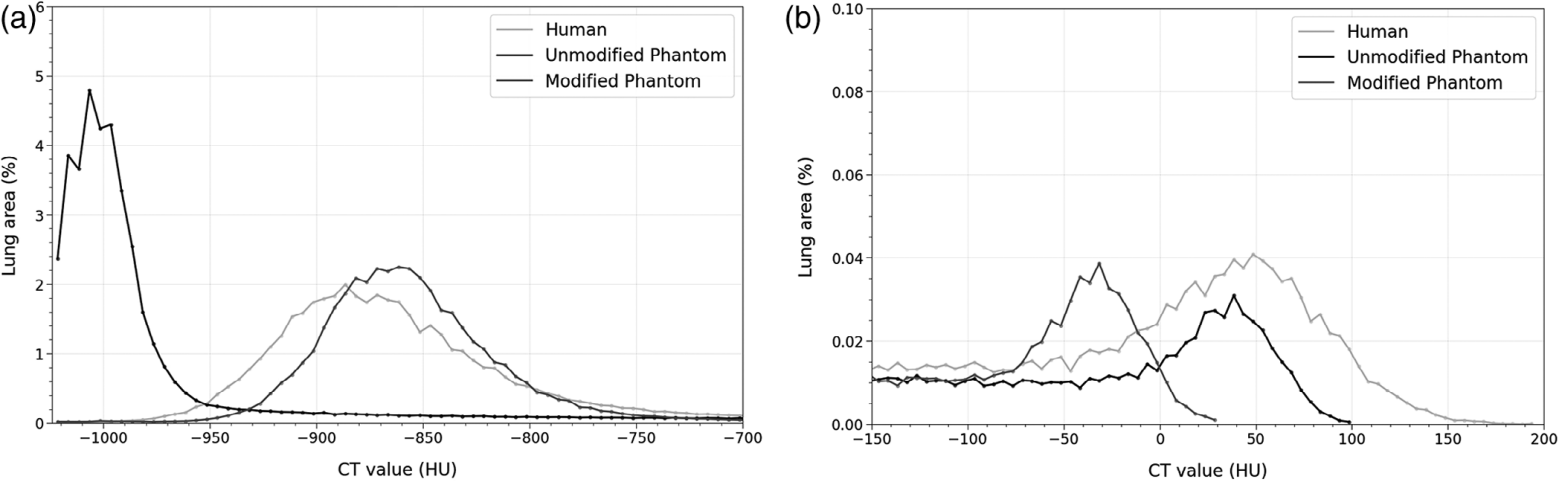
Normalized histograms (lung area percentage), split into two sections, showing CT value distributions for (a) lung parenchyma from −1024 to −700 HU and (b) bronchovasculature from −150 to 200 HU for human, unmodified, and modified phantom scans.

Qualitative assessment of the CT value distribution showed a high degree of overlap between the modified phantom and human CT scans for lung parenchyma, with maximum CT value frequencies at −862 and −887 HU, respectively. In contrast, the unmodified phantom, which lacked lung‐equivalent material, had a maximum at −996 HU (Figure 5a). For the bronchi and vasculature, the unmodified phantom showed greater overlap with the human CT scan, with maximum CT value frequencies at 39 and 51 HU, respectively. The modified phantom, however, exhibited a shift toward lower attenuation values, peaking at −31.5 HU (Figure 5b).

### 3.2 Radiodensity of pulmonary structures

**FIGURE 6:**
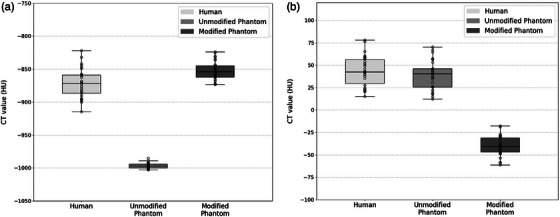
Boxplots displaying the median and interquartile range (IQR, 25th–75th percentiles) CT values for (a) lung parenchyma and (b) bronchovasculature in the human, unmodified phantom, and modified phantom CT scans, acquired at a tube voltage of 120 kVp.

Median and interquartile range (IQR, 25th–75th percentiles) CT values in the human scan were −872 (−886, −859) HU for lung parenchyma and 42 (30, 56) HU for vasculature (Figure 6). In comparison, at 120 kVp tube voltage, the unmodified phantom showed significantly lower CT values for lung parenchyma at −997 (−1000, −994) HU (*p* < 0.05), while the modified phantom's CT values were closer to human data at −854 (−862, −845) HU (*p* > 0.05). For vasculature and bronchi, CT values were 40 (26, 46) HU in the unmodified phantom (*p* > 0.05) and significantly lower at −41 (−47, −31) HU in the modified phantom (*p* < 0.05).

**FIGURE 7:**
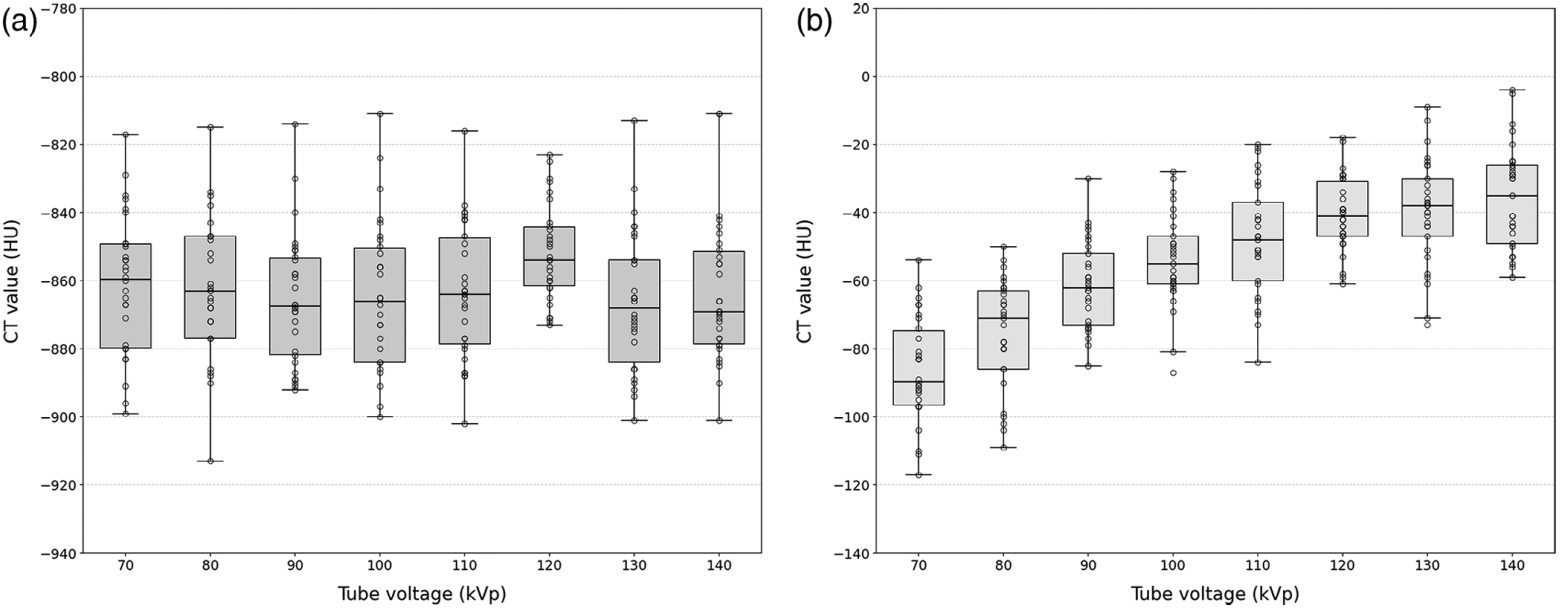
Boxplots displaying the median and interquartile range (IQR, 25th–75th percentiles) CT values measured at tube voltages ranging from 70 to 140 kVp for (a) lung parenchyma and (b) bronchovasculature of the modified phantom.

In the modified phantom, the energy dependence of measured CT values for lung parenchyma and bronchovascular materials was assessed (Figure 7). Lung parenchyma showed no significant variation in CT values across tube voltages, indicating energy independence (*p* > 0.05). In contrast, bronchovascular CT values varied significantly with tube voltage, showing a trend toward higher CT values at increasing kVp settings (*p* < 0.05).

### 3.3 Subjective evaluation

**FIGURE 8:**
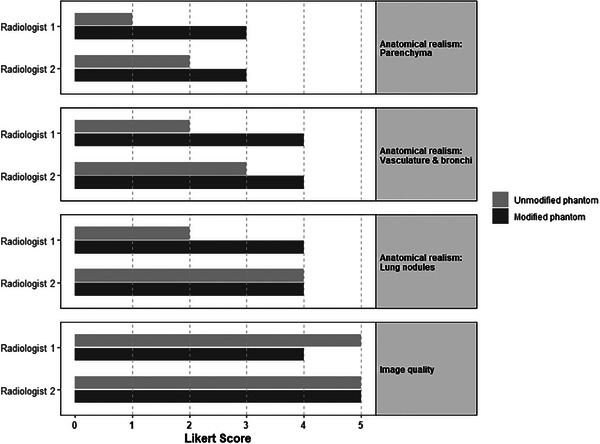
Bar chart displaying the Likert scores (1 = very poor, 2 = poor, 3 = acceptable, 4 = good, 5 = very good) for anatomical realism of lung vasculature and bronchi, parenchyma, lung nodules, and image quality, as assessed by two radiologists. The scores compare the modified and unmodified phantoms across all evaluated aspects.

The modified phantom was rated higher than the unmodified phantom in subjective evaluation, particularly for anatomical realism of lung parenchyma, brochovasculature, and lung nodule appearance (Figure 8). Across both radiologists, the modified phantom received a higher overall Likert score for anatomical realism (mean 3.7  ±  0.5) compared to the unmodified phantom (mean 2.3  ±  1.0). The image quality was rated as good to very good, with mean Likert scores of 4.5 for the modified phantom and 5.0 for the unmodified phantom.

Radiologist feedback supported these findings. In the unmodified phantom, vessels were described as overly abundant, extending too peripherally, and lacking natural tapering. Additionally, the absence of bronchi and lung parenchyma further reduced anatomical realism. In contrast, the modified phantom had bronchi of appropriate width and sufficient vasculature for evaluation, but the bronchial walls were considered too thick, abruptly terminated, and partially filled with residual 3D‐print material. Also, some parenchymal details were noted as insufficient, lacking fine linear structures such as inter‐ and intralobular septa. Regarding lung nodules, Radiologist 1 noted that in the modified phantom, nodules appeared more realistically embedded within the parenchyma, with a more natural balance between surrounding vessels and tissue. In the unmodified phantom, nodules were consistently located adjacent to large vessels, along bronchovascular bundles, or against the pleura. Radiologist 2 praised the flexibility in the modified phantom to place nodules either attached to or separate from bronchial vessels. While the modified phantom received high ratings for overall image quality, it scored slightly lower than the unmodified phantom due to “increased noise in the lung parenchyma.”

## 4 DISCUSSION

In this study, the life‐sized, patient‐specific anthropomorphic thorax phantom “Lungman” was modified to incorporate lung parenchyma and bronchi, features absent in the unmodified version. Qualitative and quantitative assessment of CT value distribution demonstrated a high degree of similarity between the modified phantom and human data for lung parenchyma, though radiodensity characteristics of the 3D‐printed bronchovascular insert could be improved. Radiologists consistently rated the modified phantom higher than the unmodified version, particularly for anatomical realism of lung parenchyma, bronchovascular structures, and appearance of lung nodules.

Several studies have presented phantoms with patient‐specific bronchial anatomy and lung parenchyma using 3D‐printing techniques.^8^ Hernandez et al. and Hong et al. developed phantoms with detailed lung anatomy and life‐like CT attenuation values.^10^, ^14^ However, these phantoms were limited to small sections of the thorax rather than a full‐scale representation. Silberstein et al. presented a 3D‐printed full‐scale phantom, but without life‐like bronchovascular anatomy.^32^ Similarly, Hazelaar et al. and Rodgers et al. introduced full‐scale 3D‐printed phantoms aimed at CT imaging quality assurance, but without internal bronchovascular features.^12^, ^17^ Lustermans et al. and Abdollahi et al. developed dynamic thoracic phantoms designed specifically for CT motion management quality assurance in radiotherapy.^33^, ^34^ While materials such as cork, foam, and synthetic 3D‐printing composites have previously been used to simulate lung parenchyma, these materials were usually molded into rigid shapes, restricting customization with lung nodules or other pathological features.^14^, ^17^ Studies on lung cancer imaging have demonstrated the benefits of using the original Lungman phantom.^22^, ^30^ However, in these studies, lung nodules had to be attached to the vasculature of the original phantom's insert or the chest wall, which does not accurately reflect the distribution of real‐life lung nodules, which are typically uniformly distributed throughout the parenchyma, often without direct contact with vascular or bronchial structures.^35^

Clinically, the modified phantom design offers a more realistic platform for CT quality assurance and protocol optimization, particularly in applications related to nodule detection and evaluation. Unlike previously reported phantoms, which are often limited to small, static sections or lack detailed internal anatomy, our model integrates full‐scale bronchial structures, tissue‐equivalent parenchyma, and interchangeable nodules within a commercially available anthropomorphic thoracic cavity. By combining anatomically realistic bronchovascular anatomy with parenchymal material, the phantom enables customizable and clinically realistic placement of lung nodules, providing a more adaptable platform for CT imaging studies related to lung cancer. While our study focused on lung nodules as pathology, the customizable design may also allow for simulation of other pulmonary pathologies. For example, as demonstrated in a study by Dunning et al., components simulating chronic obstructive pulmonary disease (COPD) or other conditions could be developed and inserted to support pathology‐specific imaging.^19^

To the best of our knowledge, this is the first study to develop a full‐scale anthropomorphic thorax phantom that combines CT‐derived 3D‐printed anatomy with lung parenchyma and nodules. The polymer (polyamide 12) used for 3D‐printing the bronchovascular insert exhibited energy‐dependent attenuation behavior, consistent with findings by Cropp et al., who reported similar characteristics in polyethylene.^36^ The cork‐based lung parenchyma showed energy‐independent CT values across kVp settings, aligning with previous findings by Brindhaban et al. and Jaafar et al. on lung tissue‐equivalent materials.^37^, ^38^

However, some aspects of the phantom could be improved. First, the 3D‐printed bronchovascular insert did not fully replicate human attenuation values. Future iterations should consider denser materials while maintaining sufficient printer resolution for fine vascular and bronchial branching details. Additionally, the printing process caused partial filling of the bronchi with melted material, which could only be partially removed during postprocessing. Second, the use of CT data from a healthy participant limited the ability to model pathological variations in bronchial and vascular anatomy. This restricts the phantom's applicability for studying conditions such as COPD and asthma, where airway morphology may be altered.^39^, ^40^ While it is possible to manually adjust the 3D‐model for printing, this process is labor‐intensive. Third, the modified phantom lacked fine parenchymal structures representing the interstitium, such as interlobular septa. Additionally, the lung parenchyma had a single uniform density, limiting its ability to simulate hypo‐ or hyperdense pathologies such as emphysema or pulmonary fibrosis. A limitation of this study is that the radiologists were informed about which images corresponded to the unmodified and modified phantom, which may have introduced potential bias in the subjective evaluations. Although this was difficult to avoid due to the radiologists' prior familiarity with the original phantom, we sought to reduce recall and comparison bias by introducing a three‐week interval between review sessions.

Addressing the aforementioned points of improvement, future work could involve optimizing material selection for 3D‐printing to achieve more accurate bronchial and vascular attenuation values while preserving fine anatomical details and preventing the unintended filling of bronchi during the printing process. Additionally, introducing morphological variations, such as thicker bronchial walls to simulate COPD or foam‐based inserts to replicate emphysematous tissue, could expand the phantom's utility for disease‐specific research.^41^ To enhance lung parenchyma realism, a potential improvement could involve layering pockets of cork granules within plastic foil compartments to simulate interlobular septa. To our knowledge, this approach has not been previously reported. Finally, to model pathologies that cause hypo‐ or hyperdense areas in the lung parenchyma, cork or foam inserts could be incorporated into the phantom.

## 5 CONCLUSIONS

This study presents a modified, patient‐specific anthropomorphic thorax phantom that integrates realistic 3D‐printed bronchial and vascular structures and lung parenchyma with lung nodules. The modified phantom demonstrates improved realism in simulating the human thorax, offering a more anatomically accurate representation of lung parenchyma, bronchovascular structures, and lung nodules.

## CONFLICT OF INTEREST STATEMENT

JFH was funded by Siemens Healthineers Nederland B.V. The remaining authors declare that the research was conducted in the absence of any commercial or financial relationships that could be construed as a potential conflict of interest.
